# Spinal anesthesia for muscle biopsy in an infant with a suspected neuromuscular disorder: a case report

**DOI:** 10.1186/s40981-020-00392-x

**Published:** 2020-10-19

**Authors:** Yoshiaki Ishida, Masato Morita, Takahisa Sasaki, Akiko Taniguchi

**Affiliations:** 1 Department of Anesthesiology, Seirei Hamamatsu General Hospital, 2-12-12 Sumiyoshi, Naka-ku, Hamamatsu-shi, Shizuoka 430-8558 Japan; 2 Department of Anesthesiology, Anjo Kosei Hospital, 28 Higashihirokute, Anjo-cho, Anjo-shi, Aichi 446-8602 Japan

**Keywords:** Neuromuscular disorder, Muscle biopsy, Spinal anesthesia, Dexmedetomidine, Infant, Regional anesthesia

## Abstract

**Background:**

Neuromuscular disorders (NMDs) occur in different forms and are generally diagnosed using muscle biopsy. Among the available anesthetic management options for infants with a suspected NMD are general anesthesia (GA) and regional anesthesia (RA), including spinal anesthesia (SA). Anesthesia selection is often challenging from the point of potential airway risks and anesthetic drug-related complications.

**Case presentation:**

A 6-month-old male infant repeatedly underwent endotracheal intubation and extubation after birth because of respiratory muscle weakness and copious secretions. He was suspected of having NMD and was scheduled for muscle biopsy. His generalized hypotonia and decreased respiratory function presented a potentially difficult airway and complicated the selection of an appropriate anesthetic method. We selected SA and dexmedetomidine, which are safe for infants.

**Conclusion:**

We report the successful and effective anesthetic management of SA and dexmedetomidine in an infant with a suspected NMD.

## Background

Neuromuscular disorders (NMDs) occur in different forms, and muscle biopsy is often required for differential diagnosis and further treatment [1]. Although general anesthesia (GA) has been predominantly performed in infants undergoing muscle biopsy, it may trigger respiratory complications, particularly in infants with decreased respiratory function due to NMD. Spinal anesthesia (SA) has been safely performed in infants [2–5] and should be an option for patients with respiratory complications. We report SA under sedation with dexmedetomidine in an infant with generalized hypotonia and decreased respiratory function undergoing muscle biopsy. Written informed consent to publish this case report was obtained from the patient’s parent.

## Case presentation

A 6-month-old male infant (weight, 4.4 kg; height, 60 cm) was born at 37 0/7 weeks of gestation with a birth weight of 2144 g. The patient’s Apgar scores were 3 and 5 at 1 and 5 min, respectively. Endotracheal intubation was performed immediately after birth by the attending neonatologist because of generalized hypotonia and insufficient respiratory function. Since he was diagnosed with neonatal asphyxia, therapeutic hypothermia was also started. Once his respiratory status stabilized, extubation was successfully performed. However, 5 days later, respiratory muscle weakness led to copious secretions resulting in pulmonary aspiration and airway obstruction, which required endotracheal reintubation. Successful extubation was finally achieved 14 days after birth. He required nocturnal continuous positive airway pressure therapy due to respiratory muscle weakness. His physical findings, such as severe hypotonia, mild arthrogryposis, and absent deep tendon reflexes informed a suspected diagnosis of NMD. To confirm this diagnosis, the attending neonatologist performed a nerve conduction velocity test, magnetic resonance imaging, and electroencephalogram, and scheduled the patient for a muscle biopsy 193 days after birth. Fortunately, the patient did not have complications such as cardiovascular disease. We decided to combine SA with dexmedetomidine because of the potential airway risk and possible anesthetic drug-related complications faced.

An intravenous catheter was inserted into the back of his hand before entering the operating room. His blood pressure was noninvasively monitored, along with electrocardiography, pulse oximetry, and continual skin temperature monitoring in the operating room. Dexmedetomidine was administered at 0.5 μg/kg for 10 min (3 μg/kg/h), followed by 0.7 μg/kg/h throughout surgery. During bolus administration, we also administered fentanyl 5 μg for analgesia and atropine 0.05 mg. Lumbar puncture was performed using a midline approach at the L4–L5 intervertebral space with a 25-gage cutting needle (25 mm) in the left lateral decubitus position because the biopsy was to be collected from the left quadriceps muscle. We slowly administered 0.18 mL/kg (0.9 mg/kg) of 0.5% hyperbaric bupivacaine.

Approximately 2 min after spinal injection, the patient was unable to move his leg and hip. Three minutes later, the sympathetic nerve block was recorded at the T11 level by loss of the pilomotor reflex. Then, the table was tilted to a reverse Trendelenburg position to avoid further cephalad spread. Anesthesia was maintained using only dexmedetomidine. For postoperative analgesia, we asked the surgeons to infiltrate the wound with 3 mL of 0.2% ropivacaine at the time of closure. He was comfortable and breathed spontaneously without additional oxygen and had stable blood pressure, pulse, and oxygen saturation throughout the 38-min surgery. Sensory and motor function gradually recovered and returned to the preoperative level at 3 h after surgery. About 2 weeks later, he was histopathologically diagnosed with myotubular myopathy.

## Discussion

NMD takes different forms and is categorized into various groups, such as M (myopathy and muscular dystrophy) and MM (mitochondrial or metabolic myopathy) [1]. A quadriceps muscle biopsy is needed to diagnose NMD, and immobilization of the lower body is required to safely perform the procedure. Anesthetic methods for biopsy include GA and regional anesthesia (RA), which includes SA. In our past experience, local infiltration anesthesia at the surgical site with sedation failed to provide adequate analgesia, and GA with volatile agents was required to prevent body movement. In this case, we combined SA under sedation with dexmedetomidine instead of using GA.

SA is generally considered to be a safe technique for children with a high success rate [2–7]. Because of the relatively large subarachnoid space and cerebrospinal fluid volume in infants compared with adults [2, 3], an anesthesiologist skilled in adult SA can easily perform it in children, as shown by lumbar puncture for children performed by pediatrician trainees. Preservative-free bupivacaine 0.5%, 0.2 mL/kg (1 mg/kg) is usually used and the duration of analgesic effect is 70 − 80 min [2]. Complications such as neurological injury and an unintentional increase in the spinal block level are extremely rare [2–5]. Moreover, in infants with a suspected NMD, RA, especially SA, rarely causes malignant hyperthermia and rhabdomyolysis, and should be used whenever possible [8, 9].

For pediatric SA, infants sometimes require analgesia and sedation in order to prevent any untoward movements during lumbar puncture [4]. We selected dexmedetomidine because it provides fast-onset sedation that parallels natural sleep, with minimal respiratory depression. Dexmedetomidine exhibits neuroprotective effects, has no known active or toxic metabolites, and has been used safely in young children [10]. Our patient achieved good analgesia and sedation with a combination of SA and dexmedetomidine during surgery.

GA should be avoided in patients with a suspected NMD because of the potential problems in securing the airway. Our patient required endotracheal intubation for pulmonary aspiration and airway obstruction due to respiratory muscle weakness and copious secretions before anesthesia. Extubation might have been difficult after GA. Unfortunately, his respiratory status was so poor that he needed a tracheostomy 2 months after this surgery. Additionally, in high-risk infants, GA has a higher incidence of postoperative apnea than SA, resulting in the risk of respiratory complications and the need for postoperative mechanical ventilation [11]. Furthermore, because GA often requires the use of volatile anesthetics and propofol, we must also be wary of rare anesthetic complications. Volatile anesthetics may induce acute rhabdomyolysis, malignant hyperthermia, and/or cardiac arrest, while propofol administration may cause acute metabolic acidosis and cardiac dysfunction in patients with a suspected NMD, including MM [1, 8].

In summary, we achieved successful and effective anesthetic management with SA and dexmedetomidine in an infant with a suspected NMD. We believe that SA is effective anesthesia because it minimizes potential airway risk in infants with a suspected NMD. Additionally, dexmedetomidine provides good sedation with minimal respiratory depression during SA. Further reports are needed to assess the feasibility and safety of combining SA and dexmedetomidine in infants with a suspected NMD.

## Data Availability

Not applicable.

## Abbreviations

NMD: Neuromuscular disorder
GA: General anesthesia
RA: Regional anesthesia
SA: Spinal anesthesia
